# Comparison of QuantiFERON-TB Gold In-Tube and QuantiFERON-TB Gold-Plus in the Diagnosis of *Mycobacterium tuberculosis* Infections in Immunocompromised Patients: a Real-World Study

**DOI:** 10.1128/spectrum.01870-21

**Published:** 2022-03-02

**Authors:** Yuzhen Xu, Qingluan Yang, Jingyu Zhou, Feiran Zhou, Yufan Hezhang, Yan Gao, Lingyun Shao, Jichan Shi, Qiaoling Ruan, Wenhong Zhang

**Affiliations:** a Department of Infectious Diseases, National Medical Center for Infectious Diseases, Shanghai Key Laboratory of Infectious Diseases and Biosafety Emergency Response, Huashan Hospital, Shanghai Medical College, Fudan University, Shanghai, People's Republic of China; b Departments of Infectious Disease, Wenzhou Central Hospital, the Dingli Clinical Institute of Wenzhou Medical University, Wenzhou, Zhejiang, People’s Republic of China; c National Clinical Research Center for Aging and Medicine, Huashan Hospital, Fudan University, Shanghai, People’s Republic of China; d Key Laboratory of Medical Molecular Virology (MOE/MOH) and Institutes of Biomedical Sciences, Shanghai Medical College, Fudan University, Shanghai, People’s Republic of China; Quest Diagnostics Nichols Institute

**Keywords:** QFT-GIT, QFT-Plus, tuberculosis, immunocompromised patients, immunocompetent patients

## Abstract

QuantiFERON-TB Gold Plus (QFT-Plus) is an emerging QuantiFERON test after QuantiFERON-TB Gold In-Tube (QFT-GIT) for tuberculosis infection detection; it is an IFN-γ release assay. We compared QFTPlus, which has an additional TB antigen 2 (TB2) tube to induce cell-mediated (CD8^+^ T cell) immune responses, with QFT-GIT. We conducted this study to assess the agreement of the QFT-GIT and QFT-Plus assays in immunocompromised patients in a clinical setting. A total of 278 immunocompromised patients and 175 immunocompetent patients from different departments were continuously enrolled from August 2020 to March 2021, and each patient underwent both tests. Correlations between QFT-GIT and QFT-Plus assays showed good agreement (κ value = 0.859). Patients receiving long-term immunosuppressant therapy had the lowest concordance between QFT-GIT and QFT-Plus assays; 9 out of 11 positive latent tuberculosis infection (LTBI) cases were diagnosed by the QFT-Plus assay, implying that QFT-Plus may detect more LTBI than QFT-GIT does in these patients. Indeterminate results were associated with lower lymphocyte, CD4^+^ T cell, and CD8^+^ T cell absolute counts, and with lower CD4/CD8 ratios. In conclusion, we found that the QFT-GIT and QFT-Plus assays had high agreement not only in immunocompetent patients but also in immunocompromised patients. QFT-Plus may detect more LTBI than QFT-GIT in patients receiving long-term immunosuppressant therapy. Thresholds were established for lymphocyte absolute counts of >1.15 × 10^9^ cells, and for CD4^+^ T cell absolute counts of >467.7 × 10^6^ to 478.5 × 10^6^ cells, which may lessen the incidence of indeterminate results.

**IMPORTANCE** This study evaluated the performance of QFT-GIT and QFT-Plus in the diagnosis of M. tuberculosis infection in immunocompromised patients and found that QFT-Plus may detect more LTBI than QFT-GIT does in patients receiving long-term immunosuppressant therapy. We believe that our study makes a significant contribution to the literature because it highlights the different diagnostic accuracies of QFT-GIT and QFT-Plus in different subpopulations of immunocompromised and immunocompetent patients. Selecting a test with better performance, particularly in patients with a high risk of developing active TB, may assist the health sector in better managing TB. Furthermore, we believe that this study will be of significance to the diagnosis of LTBI.

## INTRODUCTION

Tuberculosis (TB) is a communicable disease caused by Mycobacterium tuberculosis and a major cause of death among infectious diseases (1). Once individuals are infected with M. tuberculosis, approximately 5% will develop active disease and the remainder will have a latent TB infection (LTBI) (2, 3). Approximately 25% of the global population have an LTBI, and 5% to 15% of these subjects will develop active disease during their lifetime (4). Studies have found that the risk of progression from LTBI to active TB is higher in immunocompromised patients than in immunocompetent individuals (5, 6). Therefore, improving TB diagnosis and preventive treatment in immunocompromised individuals can largely reduce the incidence of active TB.

Currently, no available microbiological tests can identify LTBI; thus, it can only be diagnosed by immunological tests (7). Interferon-γ (IFN-γ) release assay (IGRA) detects the level of IFN-γ secreted by peripheral T lymphocytes sensitized to M. tuberculosis. IGRA reduces the false positivity rate in subjects with prior Bacillus Calmette-Guérin (BCG) vaccination or non-tuberculous mycobacteria infection compared with the tuberculin skin test (TST) (8, 9). However, IGRA does not measure the infection itself; it is a surrogate immunological test which cannot distinguish between active TB and LTBI. Notably, preventive TB treatment has no effect on the results of a QuantiFERON-TB Gold In-Tube (QFT-GIT) assay, a type of IGRA, when evaluated in a highly TB-endemic country (10).

The QuantiFERON-TB Gold Plus (QFT-Plus) assay was developed as a newer version of QFT-GIT; it contains two antigen tubes for the detection of adaptive immune responses in TB infection (11). Unlike in QFT-GIT, the TB antigen 1 (TB1) tube in QFT-Plus mainly contains peptides from the 6-kDa early secretory antigenic target and the 10-kDa culture filtrate protein, but not TB7.7, which induces CD4^+^ T-helper lymphocyte responses, while the TB antigen 2 (TB2) tube is characterized by both CD4^+^ T-helper lymphocyte and CD8^+^ cytotoxic T lymphocyte responses. CD8^+^ T cell responses are associated with active TB and recent M. tuberculosis infections (12, 13). Importantly, one study has shown that the addition of peptides for eliciting CD8^+^ T cells increases the sensitivity of testing for LTBI (14). Hence, CD8^+^ T lymphocytes can reflect the immune status of patients with few CD4^+^ lymphocytes who have been exposed to M. tuberculosis.

Several studies have compared the differences between the QFT-GIT and QFT-Plus assays in the diagnosis of M. tuberculosis infection in different populations. However, whether QFT-Plus is advantageous in immunocompromised patients remains to be determined. In this study, we analyzed the performance of QFT-GIT and QFT-Plus in immunocompromised patients in a clinical setting to provide a basic understanding of the differences between the two assays.

## RESULTS

### Clinical characteristics.

Among 482 patients, 29 active tuberculosis disease (ATB) patients were excluded, and 278 were immunocompromised; of these, 125 were undergoing long-term immunosuppressant therapy, 69 had invasive fungal infections, 56 had malignant tumors, and 28 had liver or kidney failure and were awaiting replacement therapy. The remaining 175 patients were immunocompetent. In total, there were 41 patients with positive QFT-GIT results and 32 patients with indeterminate QFT-GIT results in the immunocompromised group, and only 8 patients receiving TB-preventive therapy. The clinical characteristics of these participants are shown in Table 1. There were no statistical differences in age, sex, lymphocyte absolute counts, or CD8^+^ T cell absolute counts between the two groups. However, significant differences were observed in CD4^+^ T cell absolute counts and CD4/CD8 ratios.

**TABLE 1:** Basic characteristics of enrolled patients^*a*^

Characteristic	Patient group							*P* value
	Immunocompromised						Immunocompetent	
	Long-term immunosuppressant therapy	Malignant tumors	Invasive fungal infection	Liver/kidney failure		Total	Total	
*N*	125	56	69	28		278	175	
Sex, male: *N* (%)	55 (44.0)	38 (67.9)	48 (69.6)	17 (60.7)	158 (56.8)		99 (56.6)	0.956
Median age (*M_IQR_*)	50.0 (35.5–64.0)	60.5 (48.3–69.5)	55.0 (40.0–65.0)	48.0 (35.8–61.5)	52.0 (38.0–64.0)		54.0 (35.0–64.0)	0.837
Absolute counts								
Lymphocyte (×10^9^/L, *M_IQR_*)	1,485.0 (867.5–2115.0)	1,360.0 (752.8–1,738.0)	1,500.0 (980.0–1,775.0)	985.0 (650.0–1,638.0)	1,380.0 (890.0–1,840.0)		1,500.0 (1050.0–2,045.0)	0.088
CD4^+^ (×10^6^/L, *M_IQR_*)	502.6 (311.8–835.4)	547.8 (347.1–783.6)	572.4 (371.5–788.9)	481.9 (255.5–680.0)	519.5 (318.4–789.2)		634.7 (390.7–869.5)	0.015
CD8^+^ (×10^6^/L, *M_IQR_*)	416.1 (267.2–672.9)^*b*^	337.5 (223.4–620.8)	352.8 (266.8–506.8)	237.1 (130.6–395.4)	361.2 (241.9–568.4)		365.9 (278.8–527.7)	0.880
CD4/CD8 ratio(*M_IQR_*)	1.330 (0.805–2.045)	1.490 (1.100–2.245)	1.390 (0.880–2.320)	1.980 (1.175–2.493)	1.390 (0.930–2.160)		1.590 (1.075–2.400)	0.006

a *M_IQR_*, median and interquartile range. *P* values and Wilcoxon signed-rank were used to compare differences between the immunocompromised and immunocompetent groups.

b Result for the long-term immunosuppressant therapy group was statistically significant compared with that of the liver/kidney failure group (*P* = 0.0002, Mann-Whitney U test).

### Comparison of IFN-γ values between QFT-Plus and QFT-GIT assays.

**FIG 1:**
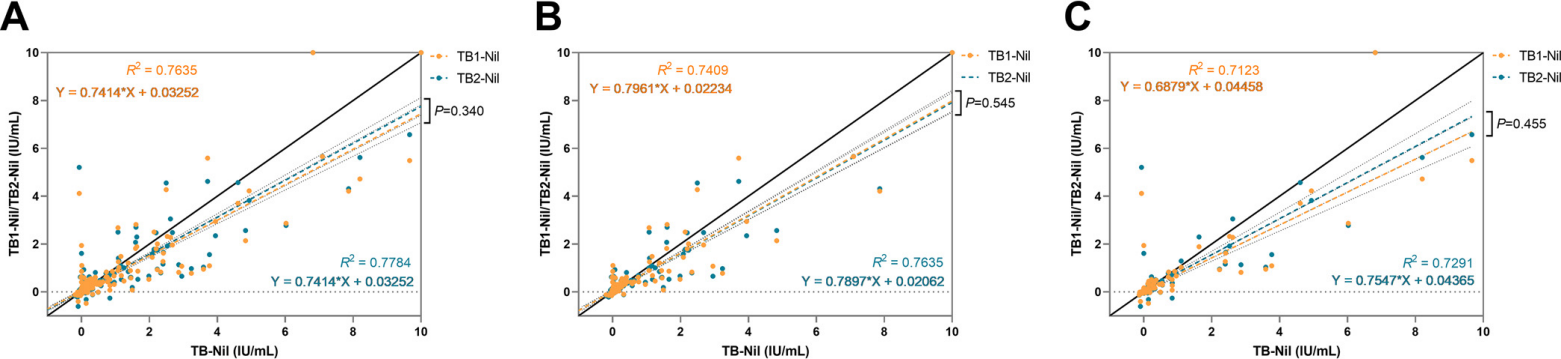
Correlation between QFT-Plus and QFT-GIT assay. (A) TB1-Nil/TB2-Nil versus TB-Nil in all patients, (B) TB1-Nil/TB2-Nil versus TB-Nil in immunocompromised patients, (C) TB1-Nil/TB2-Nil versus TB-Nil in immunocompetent patients. The correlation between QFT-Plus and QFT-GIT assays was determined using Spearman’s correlation coefficient, and the relation in IFN-γ levels between QFT-Plus and QFT-GIT were determined using linear regression analyses.

The IFN-γ levels of TB tube in the QFT-GIT assay were higher than those of TB1 and TB2 tubes in the QFT-Plus assay for all patients, but with no statistical difference. (0.368 versus 0.327, *P* = 0.276 and 0.368 versus 0.336, *P* = 0.160, respectively). Furthermore, the difference between the IFN-γ values for TB2-Nil and TB1-Nil tube was not significant for the immunocompromised group, the immunocompetent group, or for all patients combined (0.309 versus 0.312, *P* = 0.340; 0.378 versus 0.350, *P* = 0.545; and 0.336 versus 0.327, *P* = 0.455, respectively) (Fig. 1).

### Correlation between QFT results and absolute counts of lymphocytes, CD4^+^, and CD8^+^ T cells.

**FIG 2:**
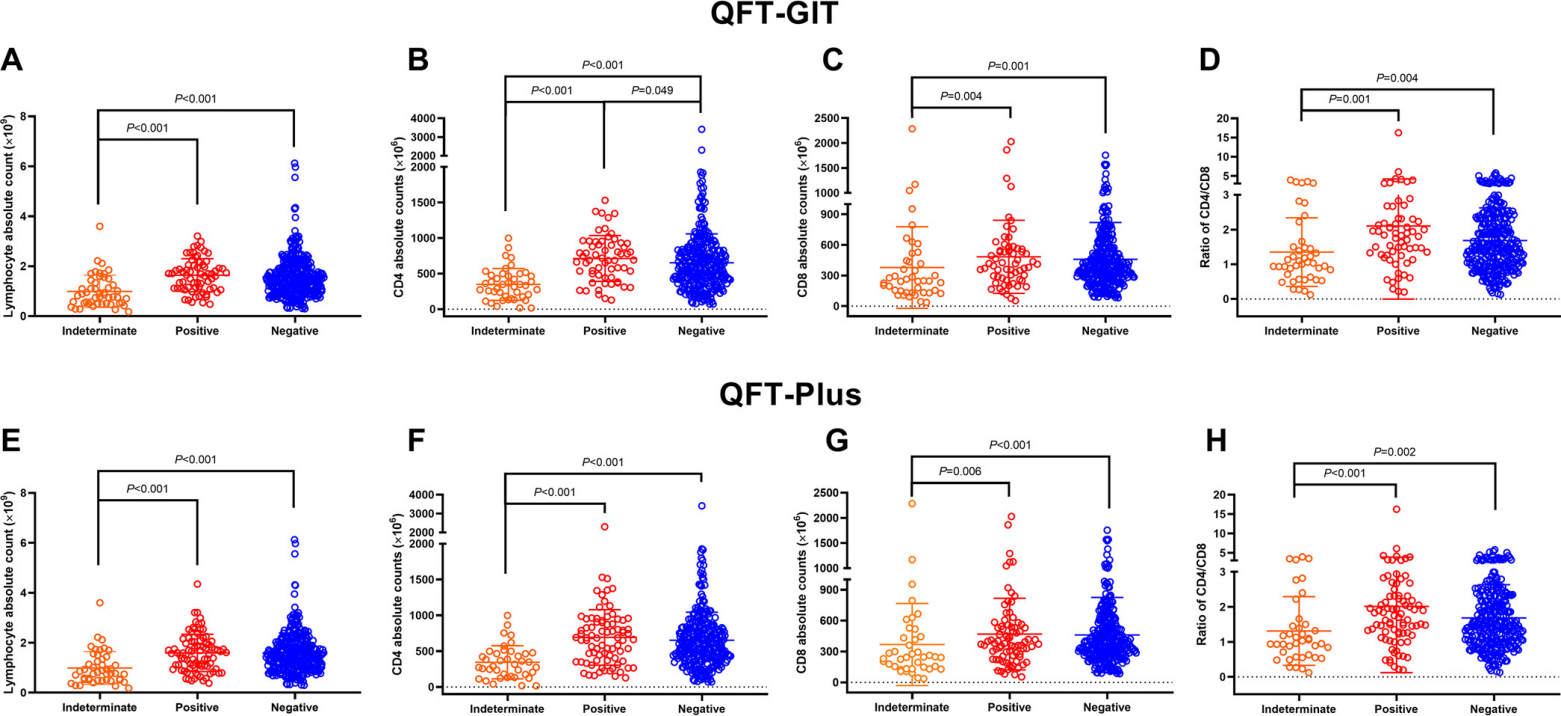
Correlation between QFT results and lymphocyte, CD4^+^ T cell, and CD8^+^ T cell absolute counts. (A) Correlation between lymphocyte absolute counts and QFT-GIT results. (B) Correlation between CD4^+^ T cell absolute counts and QFT-GIT results. (C) Correlation between CD8^+^ T cell absolute counts and QFT-GIT results. (D) Correlation between CD4/CD8 ratios and QFT-GIT results. (E) Correlation between lymphocyte absolute counts and QFT-Plus results. (F) Correlation between CD4^+^ T cell absolute counts and QFT-Plus results. (G) Correlation between CD8^+^ T cell absolute counts and QFT-Plus results. (H) Correlation between CD4/CD8 ratios and QFT-Plus results. Mann-Whitney U test for unpaired design. A *P* value of <0.05 was considered a statistically significant difference.

**FIG 3:**
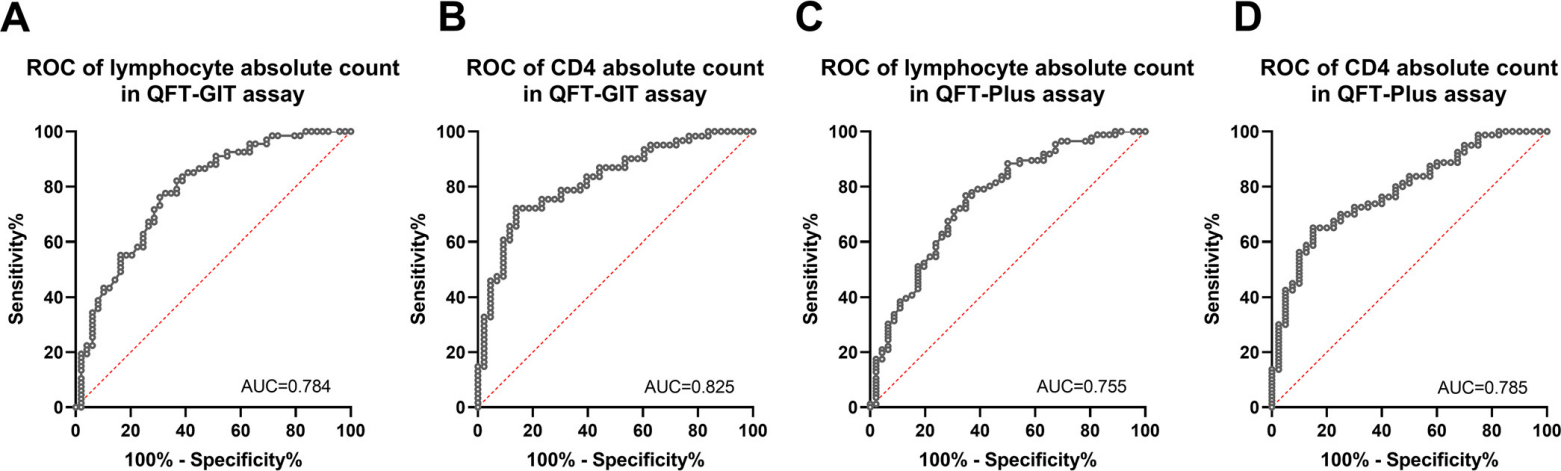
Receiver operating characteristics (ROC) curve analysis of QFT results and lymphocyte and CD4^+^ T cell absolute counts. (A) ROC curve of indeterminate and positive results in the QFT-GIT assay and lymphocyte absolute counts. (B) ROC curve of indeterminate and positive results in the QFT-GIT assay and CD4^+^ T cell absolute counts. (C) ROC curve of indeterminate and positive results in the QFT-Plus assay and lymphocyte absolute counts. (D) ROC curve of indeterminate and positive results in the QFT-Plus assay and CD4^+^ T cell absolute counts. The ROC curves were generated for discriminating indeterminate results from positive results. The areas under the curves (AUC) were assessed to evaluate performance for discriminating the indeterminate results.

All the patients were classified into indeterminate, positive, or negative groups for both QFT-GIT and QFT-Plus. The absolute counts of lymphocytes, CD4^+^ T cells, and CD8^+^ T cells, and the CD4/CD8 ratios, were significantly lower in patients with indeterminate results than in patients with positive and negative results, but the differences between the positive and negative groups were not significant except for the CD4^+^ T cell absolute counts in the QFT-GIT assay (Fig. 2). We used a receiver operating characteristic (ROC) curve analysis to determine the optimal cutoff value which reduced the indeterminate results. As shown in Fig. 3, the optimal cutoff value for lymphocytes absolute counts was 1.15 × 10^9^/L (sensitivity, 71.6%; specificity, 71.4%; 95% CI, 0.699 to 0.869), the one for CD4^+^ T cell absolute counts was 478.5 × 10^6^/L (sensitivity, 75.4%; specificity, 76.7%; 95% CI, 0.745 to 0.904) in the QFT-GIT assay, the one for lymphocyte absolute counts was 1.15 × 10^9^/L (sensitivity, 70.9%; specificity, 69.6%; 95% CI, 0.667 to 0.843), and the one for CD4^+^ T cell absolute counts was 467.7 × 10^6^/L (sensitivity, 70.0%; specificity, 75.0%; 95% CI, 0.701 to 0.868) in the QFT-Plus assay.

### Concordance between QFT-Plus and QFT-GIT.

Concordance between QFT-Plus and QFT-GIT is shown in Tables 2 and 3. The agreement rate between QFT-Plus and QFT-GIT assays was 93.8% (κ value = 0.859) in all patients, 93.8% (κ value = 0.851) in the immunocompromised group, and 93.9% (κ value = 0.863) in the immunocompetent group. Notably, 11.5% and 9.7% of the results were indeterminate when diagnosed using QFT-GIT for the immunocompromised and immunocompetent groups, respectively. There were 49 and 46 indeterminate results determined by QFT-GIT and QFT-Plus, respectively. Three of the 49 QFT-GIT indeterminate results were diagnosed as positive by the QFT-Plus assay. The difference in sensitivity between QFT-Plus and QFT-GIT in the immunocompromised and immunocompetent groups was statistically significant (χ^2^ = 443.850, *P* < 0.001; χ^2^ = 263.922, *P* < 0.001). The concordances between the IFN-γ responses of QFT-Plus TB1 or TB2 and QFT-GIT TB were relatively poorer in the long-term immunosuppressant therapy group than in the liver/kidney failure, malignant tumors, and invasive fungal infection groups (Table 3).

**TABLE 2:** Concordance between QFT-GIT and QFT-Plus^*a*^

QFT-GIT	No. (%) of concordant QFT-Plus results			
	Negative	Indeterminate	Positive	Total
All patients				
Negative	315 (69.5)	0 (0.0)	20 (4.4)	335 (73.9)
Indeterminate	0 (0.0)	46 (10.2)	3 (0.7)	49 (10.9)
Positive	5 (1.1)	0 (0.0)	64 (14.1)	69 (15.2)
Total	320 (70.6)	46 (10.2)	87 (19.2)	453 (100.0)
Immunocompetent group				
Negative	124 (70.9)	0 (0.0)	6 (3.4)	130 (74.3)
Indeterminate	0 (0.0)	15 (8.6)	2 (1.1)	17 (9.7)
Positive	3 (1.7)	0 (0.0)	25 (14.3)	28 (16.0)
Total	127 (72.6)	15 (8.6)	33 (18.8)	175 (100.0)
Immunocompromised group				
Negative	191 (68.7)	0 (0.0)	14 (5.0)	205 (73.7)
Indeterminate	0 (0.0)	31 (11.2)	1 (0.4)	32 (11.6)
Positive	2 (0.7)	0 (0.0)	39 (14.0)	41 (14.7)
Total	193 (69.4)	31 (11.2)	54 (19.4)	278 (100.0)

a QFT-GIT, QuantiFERON-TB Gold In-Tube; QFT-Plus, QuantiFERON-TB Gold-Plus.

**TABLE 3:** Concordance between QFT-GIT and QFT-Plus in immunocompromised group^*a*^

Patient groups and assays compared	No. of patients for whom assays showed agreement (%)	κ value
Immunocompromised patients (*n* = 278)		
QFT-GIT vs QFT-Plus	261 (93.9)	0.863
QFT-GIT vs QFT-Plus TB1	263 (94.6)	0.874
QFT-GIT vs QFT-Plus TB2	266 (95.7)	0.901
QFT-Plus TB1 vs QFT-Plus TB2	265 (95.3)	0.895
		
Long-term immunosuppressant therapy (*n* = 125)		
QFT-GIT vs QFT-Plus	115 (92.0)	0.810
QFT-GIT vs QFT-Plus TB1	117 (93.6)	0.837
QFT-GIT vs QFT-Plus TB2	118 (94.4)	0.866
QFT-Plus TB1 vs QFT-Plus TB2	116 (92.8)	0.813
		
Malignant tumors (*n* = 56)		
QFT-GIT vs QFT-Plus	53 (94.6)	0.899
QFT-GIT vs QFT-Plus TB1	53 (94.6)	0.896
QFT-GIT vs QFT-Plus TB2	54 (96.4)	0.932
QFT-Plus TB1 vs QFT-Plus TB2	53 (94.6)	0.899
		
Invasive fungal infection (*n* = 69)		
QFT-GIT vs QFT-Plus	66 (95.7)	0.905
QFT-GIT vs QFT-Plus TB1	66 (95.7)	0.905
QFT-GIT vs QFT-Plus TB2	66 (95.7)	0.905
QFT-Plus TB1 vs QFT-Plus TB2	69 (100.0)	1.000
		
Liver or kidney failure (*n* = 28)		
QFT-GIT vs QFT-Plus	27 (96.4)	0.873
QFT-GIT vs QFT-Plus TB1	27 (96.4)	0.873
QFT-GIT vs QFT-Plus TB2	28 (100.0)	1.000
QFT-Plus TB1 vs QFT-Plus TB2	27 (96.4)	0.873

a QFT-GIT, QuantiFERON-TB Gold In-Tube; QFT-Plus, QuantiFERON-TB Gold-Plus.

### Discordant results between QFT-Plus and QFT-GIT.

Twenty-eight discordant cases were observed (Table 4). In the immunocompromised group, there were 17 (11.5%) patients with discordant results (Table 4). Fourteen (5.0%) patients had negative QFT-GIT results and positive QFT-Plus results; 9 of these were patients undergoing long-term immunosuppressant therapy, and 4 cases were attributed to an additional TB2 antigen response in QFT-Plus. Furthermore, 1 (0.4%) subject had indeterminate results for the QFT-GIT assay and positive results for the QFT-Plus assay, while 2 (0.7%) subjects had a positive result for the QFT-GIT assay but a negative result for the QFT-Plus assay. In the immunocompetent group, 11 (6.3%) patients had discordant results; 6 (3.4%) had negative results for the QFT-GIT assay and positive results for the QFT-Plus assay, 2 (1.1%) had indeterminate results for the QFT-GIT assay and positive results for the QFT-Plus assay, and 3 (1.7%) had a positive result for the QFT-GIT assay but a negative result for the QFT-Plus assay.

**TABLE 4:** Discordant results between QFT-Plus and QFT-GIT in immunocompromised and immunocompetent groups^*a*^

Patient no.	Subgroup	Previous TB-related results	QFT-GIT		QFT-PLUS			Mitogen-Nil value
			Result	TB-Nil value	Result	TB1-Nil value	TB2-Nil value	
Immunocompromised group								
1	Long-term immunosuppressant therapy	T-SPOT.TB (+)	–^*b*^	0.304	+	0.269	0.354	2.020
2	Long-term immunosuppressant therapy		–	0.082	+	0.591	0.576	1.664
3	Long-term immunosuppressant therapy		–	0.021	+	0.224	0.405	1.794
4	Long-term immunosuppressant therapy		–	0.275	+	0.554	0.379	>10.000
5	Long-term immunosuppressant therapy		–	−0.034	+	0.029	0.622	>10.000
6	Long-term immunosuppressant therapy		–	0.070	+	0.037	0.928	>10.000
7	Long-term immunosuppressant therapy		–	0.323	+	0.371	0.253	>10.000
8	Long-term immunosuppressant therapy		–	0.000	+	0.639	0.615	0.710
9	Long-term immunosuppressant therapy		–	0.008	+	1.104	0.028	1.063
10	CKD stage 5		–	−0.014	+	0.606	0.105	2.495
11	Cryptococcal meningitis	T-SPOT.TB (+)	–	0.277	+	0.446	0.415	>10.000
12	Pulmonary aspergillosis		–	0.007	+	0.855	−0.014	>10.000
13	Malignant tumor		–	0.266	+	0.211	0.351	9.310
14	Pulmonary aspergillosis		–	0.247	+	0.424	0.448	8.453
15	Long-term immunosuppressant therapy	T-SPOT.TB (–)	Indeterminate	0.002	+	0.412	0.027	0.212
16	Long-term immunosuppressant therapy	QFT-GIT (–)	+	0.577	–	0.337	0.259	>10.000
17	Pulmonary aspergillosis	QFT-GIT (+)	+	1.495	–	0.078	0.187	>10.000
							
Immunocompetent group								
1	Blood infection		+	0.836	–	0.075	−0.261	>10.000
2	Acute interstitial nephritis		+	0.494	–	−0.011	−0.015	4.325
3	Retinal vasculitis		+	0.550	–	0.296	0.287	5.858
4	Shoulder infection	T-SPOT.TB (+)	–	0.294	+	0.399	0.342	>10.000
5	UCTD		–	0.147	+	0.443	0.265	>10.000
6	IgA nephropathy		–	0.154	+	0.303	0.369	>10.000
7	Membranous nephropathy		–	0.345	+	0.450	0.511	>10.000
8	Small intestine ulcers		–	0.296	+	0.357	0.414	5.818
9	Abnormal liver function		–	0.000	+	1.938	1.609	0.715
10	Blood infection		Indeterminate	0.231	+	0.524	0.635	0.315
11	Rheumatic heart disease		Indeterminate	−0.066	+	4.120	5.206	0.129

a QFT-GIT, QuantiFERON-TB Gold in-Tube; QFT-Plus, QuantiFERON-TB Gold Plus; CKD, chronic kidney disease; undifferentiated connective tissue disease, UCTD.

b –, Negative result; +, positive result.

## DISCUSSION

In this study, we compared the capabilities of QFT-GIT and QFT-Plus for diagnosing M. tuberculosis infection in different patients with different immune statuses in a clinical setting. The agreement between these two assays in immunocompetent and immunocompromised patients separately was high, approximately 90%. IFN-γ levels showed no significant difference between the QFT-GIT and QFT-Plus assays, but the positive result rate of QFT-Plus was significantly higher than that of QFT-GIT. In the subgroup analysis, the patients in the long-term immunosuppressant therapy group had the lowest concordance between the QFT-GIT and QFT-Plus assays, with 9 out of 14 positive LTBI cases diagnosed by the QFT-Plus assay. This result indicates that QFT-Plus may detect more LTBI than QFT-GIT assay does in these populations.

Previous studies found that the agreement between QFT-GIT and QFT-Plus was 96.6% in patients at risk for TB and in health care workers (15), 93.7% in immunocompromised patients (16), 91.1% in immunocompetent patients (17), and 86.8% in clinical samples (18). Compared with previous studies, ours is a real-world study with a relatively large sample size focused on immunocompromised patients in clinical settings, with findings that are similar to previous ones. Another study which compared QFT-Plus to QFT-GIT did not show evidence to support the superior performance of QFT-Plus in individuals with active TB and LTBI (19). QFT-Plus might be more useful for detecting LTBI in elderly and immunocompromised patients within small samples (20, 21). One study speculated that, owing to the two tubes in the QFT-Plus assay, a positive finding in either tube is considered a positive result; thus, the percentage of positive results would increase (22). Notably, a systematic review and meta-analysis found that the sensitivity of QFT-Plus was 1.3% higher than that of QFT-GIT, but the sensitivity of QFT-Plus in immunocompromised subjects requires further assessment (23). In this study, the QFT-Plus assay had a higher positive-result rate than the QFT-GIT assay in patients with two different immune statuses, and the results of the two assays showed significant differences.

Several studies found that the levels of IFN-γ in TB2 tubes were significantly higher than those in TB and TB1 tubes and that the levels of IFN-γ in TB1 tube were significantly higher than those in TB tubes (18, 24). However, the IFN-γ levels of TB, TB1, and TB2 tubes had no significant difference in our study. Other studies have also reported significantly higher median IFN-γ levels in QFT-GIT than in QFT-Plus, and suggested this could be influenced by the elimination of the TB7.7 peptide (25, 26). The IFN-γ values in TB2 tubes were higher than those in TB1 tubes in the immunocompetent group, but there were no significant differences.

One study found that the sensitivity of QFT-GIT may be reduced in patients taking immunosuppressive medications owing to the primary disease itself or to its treatment, resulting in increased false negatives (27). Analyzing the concordance of QFT-GIT and QFT-Plus in different subgroups in immunocompromised patients, we found that the κ value was the lowest for those in the long-term immunosuppressant therapy group. Further exploration showed that the more inconsistent cases in the long-term immunosuppressant therapy group were diagnosed as positive by QFT-Plus assays. It may be speculated that the group receiving long-term immunosuppressant therapy had relatively compromised T cell function and counts, but uncompromised CD8^+^ T cell response and absolute counts. We hypothesize that the QFT-Plus assay may detect more LTBI than the QFT-GIT assay in patients receiving immunosuppressant therapy, but the exact mechanisms should be explored.

The rate of indeterminate results in the immunocompromised group was higher than that in the immunocompetent group in our study. The rate of indeterminate results was 7% in controls free from glucocorticoid and immunosuppressant treatment (28), but was up to 30% in patients with HIV and in those receiving high-dose corticosteroid therapy (29, 30). Previous studies have found that lymphopenia, chronic renal disease, autoimmune disease, and chronic lung disease are independent predictive factors for indeterminate results from a QFT-GIT test (31). Inpatients in the immunocompetent group had no obvious immunocompromised factors; however, their disease situations, such as diabetes, chronic lung disease, and so on, might affect their immune status. This might explain the relatively higher incidence of indeterminate results, 9.7%, compared with previous reports. A previous study focused on patients with hematological malignancies found that the rate of QFT-GIT indeterminate results could be as high as 30.3%, associated with an abnormal white blood count and a lower median lymphocyte count (32). In our study, similar to other related studies (32 to 34), the CD4^+^ T cell absolute counts and lymphocyte counts in the immunocompromised group were significantly lower than those in the immunocompetent group. We also found that patients with indeterminate results had lower lymphocyte absolute counts, CD4^+^ and CD8^+^ T cell absolute counts, and CD4/CD8 ratios. This means that the indeterminate results were correlated with lymphocyte levels and with CD4^+^ and CD8^+^ T cell absolute counts. We used ROC curves to characterize the prognostic relevance of indeterminate results. Thresholds were established for lymphocyte absolute counts of >1.15 × 10^9^ cells, and for CD4^+^ T cell absolute counts of >467.7 ×_ _10^6^ to 478.5 × 10^6^ cells. These findings could guide physicians to determine the optimal time to implement the QFT test, making uninterpretable indeterminate QFT results less likely.

Our study has some limitations. First, we did not carry out the T-SPOT.TB or TST assays at the same time, as a reference standard to judge the accuracy of the QFT-GIT and QFT-Plus assays. Therefore, it is impossible to estimate whether the inconsistency between QFT-GIT and QFT-Plus results is due to operational error or to objective differences. Second, we failed to clarify the mechanism by which the QFT-Plus assay could improve LTBI diagnosis in patients receiving long-term immunosuppressant therapy. Hence, studies including larger samples and dividing different groups according to immunosuppressant category, dosage, and course are needed for verification.

In conclusion, we found that QFT-GIT and QFT-Plus assays have high agreement, not only in immunocompetent patients but also in immunocompromised patients. QFT-Plus may detect more LTBI than QFT-GIT in immunocompromised patients, especially those receiving long-term immunosuppressant therapy, but further studies are needed. Thresholds were established for lymphocyte absolute counts, >1.15 × 10^9^ cells; and for CD4^+^ T cell absolute counts, >467.7 × 10^6^ to 478.5 × 10^6^ cells, which may lessen the incidence of indeterminate results.

## MATERIALS AND METHODS

### Study population.

We prospectively recruited consecutive patients referred for *M. tuberculosis* infection screening at Huashan Hospital, which is affiliated with Fudan University, from August 2020 to March 2021. The enrolled patients were obtained from the infectious disease, rheumatology, hematology, oncology, and nephrology departments. Active tuberculosis disease (ATB) patients were diagnosed by clinical examination, chest computerized tomography scan, and microbiological examination (sputum smear, culture, and/or GeneXpert MTB/RIF), and these patients were excluded. After ATB patients were excluded, the remaining patients were divided according to immune status into an immunocompromised group and an immunocompetent group. The immunocompromised group mainly included patients undergoing long-term immunosuppressant therapy (including treatment with anti-tumor necrosis factor agents, cytotoxic drugs, or steroids for >2 months), those with malignant tumors (including solid tumors and hematological malignancies), those with invasive fungal infections, and those with end-stage liver disease (ESLD, with decompensated cirrhosis or liver failure) or end-stage renal disease (ESRD, estimated glomerular filtration rate of < 15 mL/min) who were awaiting replacement therapy. The immunocompetent group included patients who were excluded from the immunocompromised group. We reviewed the patients’ medical history and laboratory examination for data analysis. The study was approved by the Huashan Hospital, affiliated with the Fudan University Ethics Committee.

### QFT-GIT and QFT-Plus.

QFT-GIT (Qiagen, Germany) and QFT-Plus (Qiagen, Germany) assays were performed according to the manufacturer’s instructions, at the same time and place, and by the same person, to minimize variables. Briefly, blood samples from all patients were collected and transferred to TB, TB1, and TB2 antigen tubes, Nil tubes, and mitogen tubes, and were immediately incubated at 37°C for 18 h. After centrifugation, an enzyme-linked immunosorbent assay (ELISA) for IFN-γ was performed simultaneously for both tests. IFN-γ levels (IU/mL) in TB, TB1, TB2, and mitogen tubes were adjusted after subtracting Nil tube levels, and were expressed as TB-Nil, TB1-Nil, TB2-Nil, and Mitogen-Nil, respectively. When the IFN-γ values of TB-Nil, TB1-Nil, or TB2-Nil were ≥0.35 IU/mL and ≥25% of the Nil value, the result was considered positive, and the reverse was true for negative results. If the IFN-γ value of the Nil tube was >8.0 IU/mL or that of the mitogen tube was <0.5 IU/mL, the result was considered indeterminate.

### Statistical analysis.

All data were analyzed using IBM SPSS Statistics (version 23.0) software (IBM corporation, Armonk, NY, USA) and GraphPad Prism 8.0 (GraphPad Software Inc., San Diego, CA, USA). Consistency among the QFT-Plus and QFT-GIT assays was evaluated using Cohen’s κ values. A chi-square test was used to determine differences between the QFT-Plus and QFT-GIT assays. Receiver operating characteristic (ROC) curves were generated for discriminating the indeterminate results from the positive ones. The areas under the curves (AUC) were assessed to evaluate their performance for discriminating indeterminate results. The correlation between QFT-Plus and QFT-GIT assay results was determined using Spearman’s correlation coefficient, and the relation in IFN-γ levels between QFT-Plus and QFT-GIT was determined using linear regression analyses. The differences between continuous variables were analyzed using the Wilcoxon signed-rank test for pairwise comparisons and the Mann-Whitney U test for unpaired design. A *P* value of <0.05 was considered statistically significant.
